# Different Markov chains modulate visual stimuli processing in a Go-Go experiment in 2D, 3D, and augmented reality

**DOI:** 10.3389/fnhum.2022.955534

**Published:** 2022-11-21

**Authors:** Carlos Andrés Mugruza-Vassallo, José L. Granados-Domínguez, Victor Flores-Benites, Luz Córdova-Berríos

**Affiliations:** ^1^Escuela Profesional de Medicina Humana, Universidad Privada San Juan Bautista (UPSJB), Lima, Peru; ^2^Facultad de Ingeniería y Arquitectura, Universidad de Lima, Lima, Peru; ^3^Universidad de Ingeniería y Tecnología (UTEC), Lima, Peru

**Keywords:** augmented reality, cognitive neuroscience, decision task, environment design, Go-Go task, Markov chain, neurocomputational models, video games

## Abstract

The introduction of Augmented Reality (AR) has attracted several developments, although the people’s experience of AR has not been clearly studied or contrasted with the human experience in 2D and 3D environments. Here, the directional task was applied in 2D, 3D, and AR using simplified stimulus in video games to determine whether there is a difference in human answer reaction time prediction using context stimulus. Testing of the directional task adapted was also done.

**Research question:** Are the main differences between 2D, 3D, and AR able to be predicted using Markov chains?

**Methods:** A computer was fitted with a digital acquisition card in order to record, test and validate the reaction time (RT) of participants attached to the arranged RT for the theory of Markov chain probability. A Markov chain analysis was performed on the participants’ data. Subsequently, the way certain factors influenced participants RT amongst the three tasks time on the accuracy of the participants was sought in the three tasks (environments) were statistically tested using ANOVA.

**Results:** Markov chains of order 1 and 2 successfully reproduced the average reaction time by participants in 3D and AR tasks, having only 2D tasks with the variance predicted with the current state. Moreover, a clear explanation of delayed RT in every environment was done. Mood and coffee did not show significant differences in RTs on a simplified videogame. Gender differences were found in 3D, where endogenous directional goals are in 3D, but no gender differences appeared in AR where exogenous AR buttons can explain the larger RT that compensate for the gender difference. Our results suggest that unconscious preparation of selective choices is not restricted to current motor preparation. Instead, decisions in different environments and gender evolve from the dynamics of preceding cognitive activity can fit and improve neurocomputational models.

**GRAPHICAL ABSTRACT:**
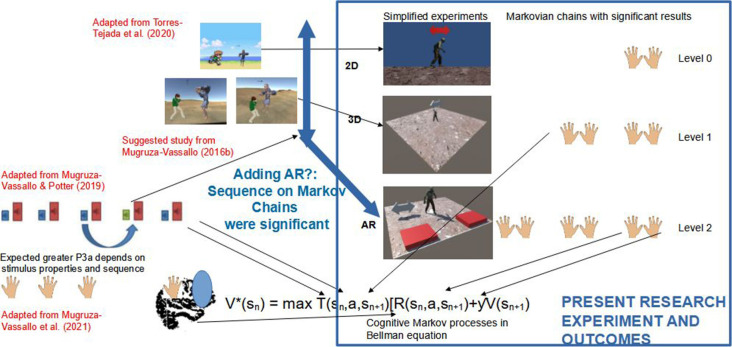


## Introduction

In the modern world, 3D stimuli is now commonplace, with most people having experienced it through education or entertainment in the form of 3D TV, as well as 3D and 2D stimuli on 2D screens. More recently, augmented reality (AR) has become more common, now being one of the main ways to receive information and in videogame controlling (e.g., DeMarle, 2017). The AR of incoming stimulus has a role in memory and cognitive load, but how AR changes memory is not clear (Schneider et al., 2020). In this study, how attention and the previous response manage AR were addressed by means of stimulus in 2D, 3D, and AR environments.

**Figure 1:**
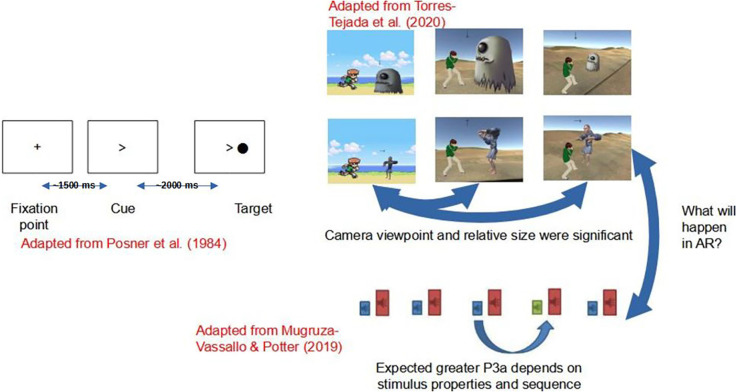
Experimental screens similar to Posner et al. (1984); Torres-Tejada et al. (2020); and Mugruza-Vassallo and Potter (2019). Those works allow us to make a simplified video-game experiment and also to ask: Are 2D, 3D, and augmented reality (AR) main differences processed able to predict with Markov chains?

The Posner paradigm of Posner permits the cost and benefit of reaction times (RTs) to be quantified (Posner, 1980), in the order of 80% valid goal stimulus in events, stimulus was presented in the area of the screen indicated by a warning signal (arrow). In 1984, this paradigm was used to study the RTs of people with damage to the parietal cortex with a greater difference observed in contralateral and ipsilateral in right parietal patients (Posner et al., 1984). Later, Posner’s paradigm was used to study task responses via ocular saccadic movement or by the pressing of a keyboard button as seen in Figure 1. The Posner paradigm has also demonstrated that microsaccades can be used to map visual orientation. This is due to the amplitude of the saccadic movement and showed RTs being higher for disabled stimuli compared to neutral stimulus or even children (Engbert and Kliegl, 2003). Moreover, Posner and colleagues have shown neuroimaging differences according to the RTs in the Attention Network Task (ANT; Fan et al., 2005) where alerting was found mainly on fronto-parietal and thalamic activation. Orienting has also been found in the parietal areas and in the frontal eye fields (FEF) and executive control has been shown to activate the Anterior Cingulate Cortex (ACC) along with other areas. An auditory version of this paradigm found a similar and complementary activation of frontal areas changing with the difference between conditions and the previous condition as well (Mugruza-Vassallo et al., 2021). This means, the warning signal in visual studies has shown real outcomes from the analysis done by cognitive human computation. The present research removed this possible covariate with a simplified version of an arrow in a videogame in 2D, 3D, and AR.

Recently, Go-Go experiments and variants of Posner’s paradigm such as ANT, coupled with tested memory, found that there was not necessarily any change in attention after the first episode of mania. However, it is possible to change other cognitive measures such as IQ, processing speed, memory work, verbal fluency, and certain executive functions (except for Go/No-Go tasks) with *p* < 0.05 (Daglas et al., 2017). Menozzi et al. (2007) tested AR stimuli of 200, 300, and 600 ms duration to locate the visual stimulus “3” in a sequence of numbers and interpreted this to mean that localisation depends on the ability to focus attention to one stimulus in an undetermined time after the signal or arrow was sent (Posner, 1980). Moreover, at the time of drawing up this manuscript a patent application has been made for an invention that relies on the benefit of the reaction time of Posner’s paradigm (1980) to improve the virtual reality teaching environment using warning signals for the adjustment of the screen, which has the advantage of attracting the attention of the user (Shahal, 2018). Alternatively, Torres-Tejada et al. (2020) developed videogames and showed for five types of visual stimuli (four types of Go-Go events, see adaption of their screens in top left part of graphical abstract). They found that the angle of presentation and the relative size of the stimulus were significant for computations between 2D and 3D. Torres-Tejada’s videogames used arrows as a guide in a similar way to the Posner paradigm. In this work, the hypothesis posed asks: is it possible to simplify the number of stimuli in a videogame and does the Go-Go task allow you to study the difference between 2D, 3D, and AR stimuli?

Therefore, in the present research, AR was compared to 2D and 3D to assess selective attention by way of reaction times (e.g., around 1,000 ms) stimuli whereas the original version of the paradigm of Posner places stimuli of alert by arrow (called endogenous signals) prior to the presentation of goal signals.

Regarding the technical part, some AR trials have used a combination of AR Tool Kit, Visual Studio C++, 3D Max, and Adobe Illustrator tools (Chen et al., 2015). In this work the previous use of Unity 3D and Vuforia made by Torres-Tejada et al. (2020) was extended from 2D and 3D stimuli to AR stimuli. Therefore, part of the technical challenge is to achieve a reasonable resolution time in data acquisition that allows measurement of the reaction times in UNITY 3D.

Taraghi and colleagues studied the dependence of the immediately preceding answer in a sequence of questions with Markov chains. The response data was acquired through an application called “1 × 1 trainer,” a tool designed for elementary school children to learn multiplication. After analysis of response times, a Markov chain of order five confirmed the probability of response on 442,910 questions answered by 3,381 children over 11,711 sessions. Some children did not take the questions seriously and other children left before the test was over (Taraghi et al., 2014). Consequently, the present work considered adult participants and therefore, the Markov chains were expected to be of an order less than five across the different presentations of 2D, 3D, and AR.

The formula (1) was used to calculate the different types of responses, where *P* is the probability and *X* the random variables were defined according to the expression (1) given as:


(1) $$P\left(X_{n+1}=x_{n+1}|X_{1}=x_{1},X_{2}=x_{2},X_{n}=x_{n}\right)=P\left(X_{n+1}=x_{n+1}|X_{n}=x_{n}\right)$$

We assume that the probabilities do not change as a function of time, hence the Markov chain transitions are time-homogeneous (Busemeyer and Townsend, 1992). Therefore, a Markov chain of order *k* is described formally as (2) used by Taraghi et al. (2014).


(2) $$P\left(X_{n+1}=x_{n+1}|X_{1}=x_{1},X_{2}=x_{2},X_{n}=x_{n}\right)=P\left(X_{n+1}=x_{n+1}|X_{n}=x_{n},X_{n-1}=x_{n-1},...,X_{n-k+1}=x_{n-k+1},X_{n}=x_{n}\right)$$

Behavioural models of goal stimuli have been used as Markov decision models (Savalia et al., 2016) or even response models for linguistic or musical events (Forth et al., 2016). Limitations in both models were that they did not consider cases of similar tasks in different modalities, moreover not citing or giving examples of cognitive responses in those models. In the present experiment and interpretation, we seek to indicate to what extent the Markov chain fits the response to events in 2D, 3D, and AR.

Conversely, the data analysis of RTs is commonly used in the Analysis of Variance (ANOVA) of the data (e.g., Potter et al., 2001; Mugruza-Vassallo, 2016a). Furthermore, other recording methods, for visual cues with letters along with results in functional Magnetic Resonance Imaging (fMRI) by Koechlin et al. (2003), Electroencephalography (EEG; Mugruza-Vassallo and Potter, 2019) and auditory fMRI results (Mugruza-Vassallo et al., 2021) have suggested that the context of the immediately preceding stimulus is significant and modulate the measurements of cognitive outcomes by recruiting a larger brain area when motor response changes between action and no-action, depending of previous novel response as a kind of Bayes analysis. Results from preceding stimuli were observed in the lower part of Figure 1, where the EEG dependence was found in the correlation of the properties of the current and the immediately prior stimuli. Moreover, prior research has found no evidence of any deficiency in N100 type potentials (Mugruza-Vassallo, 2016b; Mugruza-Vassallo and Potter, 2019) in agreement with Rosburg (2018). Likewise, the research of Samrani et al. (2018) also involved the context of working memory relating to stimuli. The previous research considered the dependence of the previous answer taken here as a possible Markov process, the present research considers a subset of characteristics to simplify the videogame compared with the case of Torres-Tejada et al. (2020), where five types of stimuli were used (see upper part of Figure 1). The present work aims to study the dependence of previous stimulus using Markov chains using only few types of stimuli in graphic format. Therefore, here it was postulated that the selective attention can be studied with responses based on an experiment which add and simplifies works of: (a) top Experiment 1: (Engbert and Kliegl, 2003), which timed three different screens (a fixing cross, an alert signal with a right arrow and a target to which the participants responded); (b) middle: 2D and 3D experiments showing RTs results altered due to stimulus properties (viewpoint and relative size); and (c) bottom: Auditory sequence with several types of stimulus has shown a strong dependence of sound properties and sequence.

Bearing in mind all the literature, the research question aimed to answer whether the Markov chains could explain occurrence states for 2D, 3D, and AR environments. Moreover, the hypothesis states that the Markov chain method in the experiment can predict the variance in those environments (2D, 3D, and AR) using the RT data. Therefore, the aim was to identify which of the 2D, 3D, and AR environments showed better attention outcomes to a simplified visual decision task by measuring accuracy and RTs, and then using Markovian reasoning to decipher the results. The question was addressed by adopting a Go-Go-type experiment which displayed an arrow that pointed either left or right to simplify the videogame stimuli.

## Material and Methods

### Participants

The experiment was carried out using 22 participants (six women) in the morning, aged from 18 to 24 years old (mean = 21.3 standard deviation = 1.1 years). Participants signed an informed consent according to the University ethical rules. Three selection criteria were used to choose participants: motivation to take the test, a general experience of playing videogames and declared that they had no impairments, being under pressure or stress that might affect their ability to pay due attention to the videogame.

### Experiment

**Figure 2:**
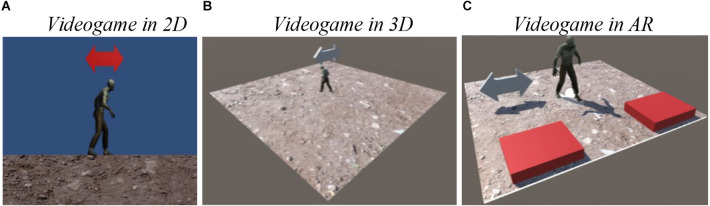
Simplified videogame using arrows on **(A)** 2D, **(B)** 3D, and **(C)** AR. The arrow was either to the left or to the right.

The software used to develop the videogame and acquire RT data was Unity 3d software (2004), a high level graphics engine that allowed us to develop three videogames for different platforms through an editor and a scripting language. To make the AR video, Vuforia complement was used (Vuforia, 2012), a useful tool to build applications based on augmented reality (e.g., Imbert et al., 2013). This is a widely used complement of software, used for computer games and mobile. The development of the present experiment’s application for 2D, 3D, and AR environments respectively, is shown in Figures 2A–C, where the same moving caricature is presented at three different environments.

**Figure 3:**
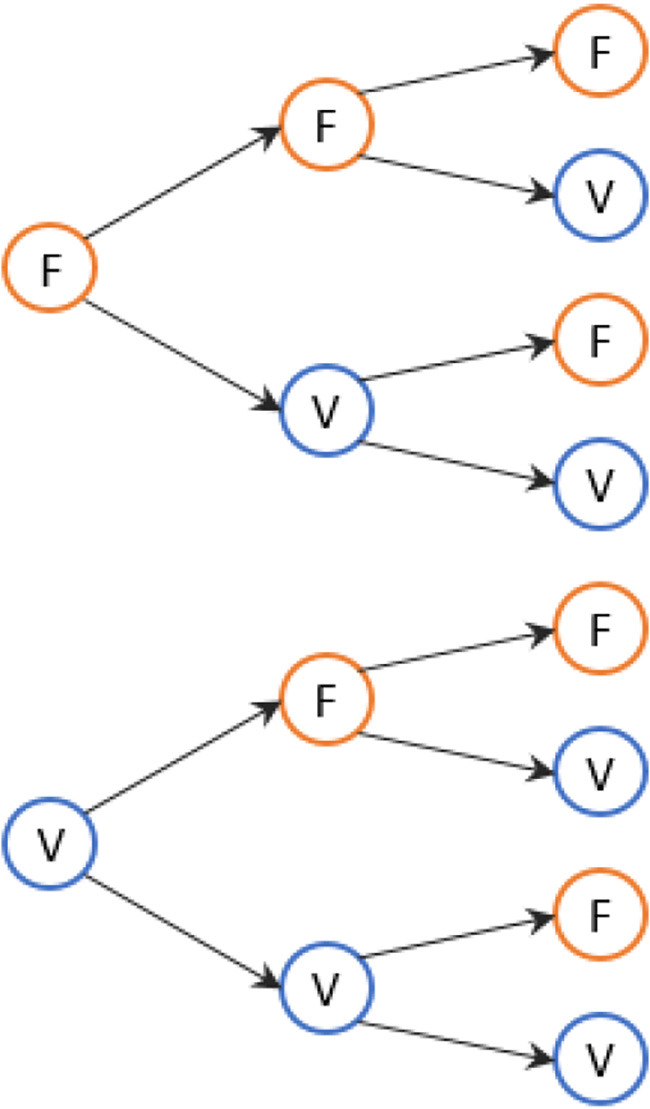
Markov chain to analyse reaction time (RT) for three consecutive stimuli.

In the experiment 120 stimuli were presented per block (360 in total). Each event at each block game started by displaying the arrow pointing either left or right for an average of 1 s, as shown in Figure 3. A random and variable time of between 0.7 s and 1.3 s was used to display the next stimulus. The participant had to identify which way the arrow was pointing and press the button to his right or left, as appropriate. Each participant was sitting down in front of a desk and maintaining head position at a viewing distance of approximately 60 cm.

### Data acquisition

RT in milliseconds (ms) were recorded, RT data were extracted whenever the user physically pressed the keyboard buttons (2D, 3D) or the virtual button (AR with the support of Logitech 922c videocamera and screen), according to the video game. RTs were measured from the moment when the arrow appeared until the virtual/physical button was pressed. Recorded data were based on the keys pressed, the participant’s RT and whether or not the correct key had been pressed, which was recorded as “true” (V) or “false” (F) state.

### Data analysis

All RTs less than 200 ms were filtered out because according to Welford (1988) this is the amount of time taken for a person to receive and process a signal. Therefore, the stimuli detection processing time is less than 200 ms, being around 170 ms the case of the human face (e.g., Rousselet et al., 2008). Therefore, the probabilities of the various RTs of response types were analysed throughout the experiment using the Markov statistical method. Finally, RTs obtained when going from one state *n* to another state *n* + 1 according to the Markov chain were analysed in detail.

Following Equation (2), the first order Markov chain (*k* = 1) and second order (*k* = 2) were described.

A Markov chain of order *k* = 1 is described formally as follows:


(3) $$P\left(X_{n+1}=x_{n+1}|X_{1}=x_{1},X_{2}=x_{2},X_{n}=x_{n}\right)=P\left(X_{n+1}=x_{n+1}|X_{n}=x_{n},X_{n-1}=x_{n-1},...,X_{n}=x_{n}\right)$$

A Markov chain of order *k* = 2 is described formally as follows:


(4) $$P\left(X_{n+1}=x_{n+1}|X_{1}=x_{1},X_{2}=x_{2},X_{n}=x_{n}\right)=P\left(X_{n+1}=x_{n+1}|X_{n}=x_{n},X_{n-1}=x_{n-1}\right)$$

The states, through which the participants were: right answer at state V (correct) and wrong answer at state F (wrong). The Markov chain used for three consecutive stimuli is seen in Figure 3.

### Markov chain for reaction times

To obtain an order 3 Markov chain from the recovered data, a State Matrix was assembled. The recovered data was then coded: “1” representing correct answers and “0” representing incorrect answers. As a result, we have a matrix of states, as represented in Table 1. This allows a quantification of the occurrences for each Markov chain and to completely build the Markov chain.

**Table 1:** Matrix of states.

0	1	1	
1	1	1	
1	1	1	
1	1	1	
1	1	1	
1	1	1	
1	1	0	
1	0	1	
0	1	1	
…	…	…

*In the Chain of States corresponding values were set at 0’s and 1’s for stimulus correctly or incorrectly answered. Each row is equivalent to three consecutive stimuli in respect to the 120 used per each participant. For example, the first chain of stimulus 1–3, second chain, 2–4, and so on*.

Finally, we converted the 0’s and 1’s to base 10 and added each chain of states formed. In analysing the three consecutive states from the 120 stimuli collected, we obtained 118 chains of states per game block made. These data represented the information needed for the final state and from this we begin to back up, in order to obtain the probabilities.

The same process was carried out for each block and for each participant and a matrix of all the videogames was assembled and afterwards a Markov chain was made per each video game.

### ANOVA of prior state RTs

The normality of the RTs was evaluated using the Shapiro-Wilk test of normality. It was found that the assumption of normality was valid. Also each RTs distribution was plotted in a histogram, no bimodal distribution was found. In order to determine whether the previous state had an influence on the current state, an ANOVA analysis was carried out on bases of the RTs of the previous states (Markov chain) and the visual environments (i.e., 2D, 3D, and AR).

Variables that might have had an effect RT, namely the influence of coffee consumption, mood and gender were also analysed with ANOVA.

## Results

### Markov chain results

**Figure 4:**
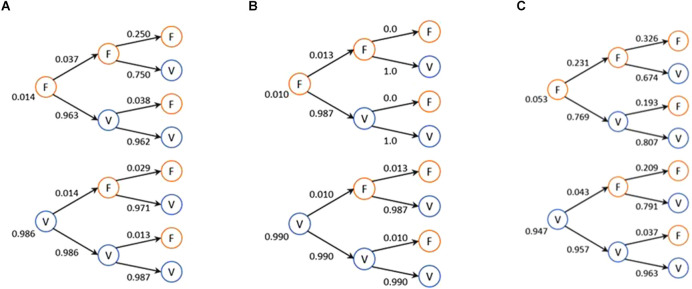
States in the AR game for all participants **(A)** in the 2D game f. **(B)** In the 3D game. **(C)** In the AR game.

In order to consolidate the data, a Markov chain was created for each video game containing the data of all the participants, as they are presented in Figures 4A–C, reflecting their true (V) and false (F) answers.

Figure 4A represents the Markov chains formed for the 2D. If the initial state is “FF” then probabilities have increased for the current state “F” (relatively with respect to the expected 12.5%). This means that if the person fails twice consecutively, the next state has a 25% chance (double the expected) of getting it wrongly and 75% of performing it correctly. If the initial state is “FV,” the probability has changed to 96.2% for participants performing the experiment correctly at the current state. If the case is “VF” or “VV,” probability of failure is very low and a large part of the probability that the answer is in the “true” state.

With respect to the Markov chain representing the 3D game (illustrated in Figure 4B), when a previous state of “FF” or “FV” was presented, there is zero probability of performing the following stimulus erroneously and instead, 100% probability of performing it correctly. In the case of “VF” and “VV,” very low probabilities were perceived, similar to what was seen in the 2D video game chain.

Finally, in Figure 4C, we recorded a large variation in probabilities. Evidently, the probability of error is greater than that in the 2D and 3D Markov chains; however, if the previous state was “VV,” the evidence suggests that there is a large probability of correctly performing the next stimulus. Additionally, if the previous state is “F” in the AR, the probability of performing the subsequent stimulus well is 76.9%, which is, however, less than the probability shown in Figure 4B (2D at 96.3%) and Figure 4C (3D at 98.7%).

### Prior state influenced RTs

Table 2 shows the ANOVA results for the participant response states (V: true, F: false) at different environments (2D, 3D, and AR). Three cases were analysed: a Markov model that considers only the current state, a Markov model that considers the current and previous state, and a Markov model which considers the current state and two previous states. In any of these models, the current state is level 0, the previous state is level 1, and so on.

In Table 2; the 0–1 levels show an influence on RTs in the two-state model. This was also seen in the three-state model, with levels of 0–2. Previous states had a significant influence (*p* < 0.05) in the RT of the current state.

**Table 2:** ANOVA effect of the current and the previous state in the reaction time.

States	Level	2D game		3D game		AR game	
		F	p	F	p	F	p
1	0	6.2	**0.0169**	1.7	0.1940	2.8	0.1034
2	0	46.3	**0.0**	49.8	**0.0**	19.4	**0.0**
	1	36.3	**0.0**	36.1	**0.0**	3.3	0.0716
3	0	57.0	**0.0**	103.5	**0.0**	13.1	**0.0004**
	1	72.0	**0.0**	88.6	**0.0**	6.6	**0.0110**
	2	71.7	**0.0**	117.7	**0.0**	59.8	**0.0**

*Results of the ANOVA analysis of the effect of the current state (level 0 for any state) and the previous ones (levels 1 and 2 for any state) in the reaction time. Significant ANOVA results in bold*.

### Coffee, mood, and gender in RTs

The analysis also considered the effect of other cofactors that might have affected the true or false responses of the participants. These were the effects of coffee consumption, mood, and gender on RTs were analysed using ANOVA.

No evidence was found that coffee consumption or mood were correlated with RT (see Table 3). However, coffee consumption is a possible study limitation that was not controlled. Previous experiments had considered excluding participants who drink more than 10 cups of coffee or had consumed coffee 3 h prior to the experiment (e.g., de Bruin et al., 2004), although other studies deprived coffee consumption (Chen et al., 2021). Furthermore the present study did not consider consecutive days of coffee administration as other studies on different coffee administrations suggested (Judelson et al., 2005) and we also did not make a comparison based on healthy and vulnerable populations with cardiovascular or sleep impairment (Temple et al., 2017).

**Table 3:** ANOVA considering answer, coffee, mood, and gender.

	Videogame 2D		Videogame 3D		Videogame AR	
	F	p	F	p	F	p
Answer (V/F)	**5.8553**	**0.02**	2.1057	0.155	2.693	0.109
Coffee	0.1688	0.684	0.7073	0.406	0.057	0.812
Mood	0.0433	0.836	0.0188	0.892	0.006	0.938
Gender	0.5396	0.467	**10.475**	**0.003**	1.798	0.188

*Significant ANOVA results in bold*.

Conversely, the 3D game introduced a different range of significant characteristics in RT between men and women (*p* = 0.003 @ *F* = 10.475). This result agreed time was interpreted shorter by women at different visual spatial angles (of 25°) in a modified Posner cueing paradigm (Cooney et al., 2017). More interesting, errors in True or False answers were found to be significant in 2D (*p* = 0.02 @ *F* = 5.8553). Supporting these results, it is believed motor control compensated for gender on the AR experiment.

Finally, bearing in mind gaming as a meaningful and purposeful activity for gamers, and as an extension of the present work, it may be useful to consider including depth mood evaluation questions, as in Shi et al. (2019) who recently found there to be caused by participants understanding phrases, in different meaning tasks (trait implying vs. control phrases).

### Improving Markovian brain modelling

**Figure 5:**
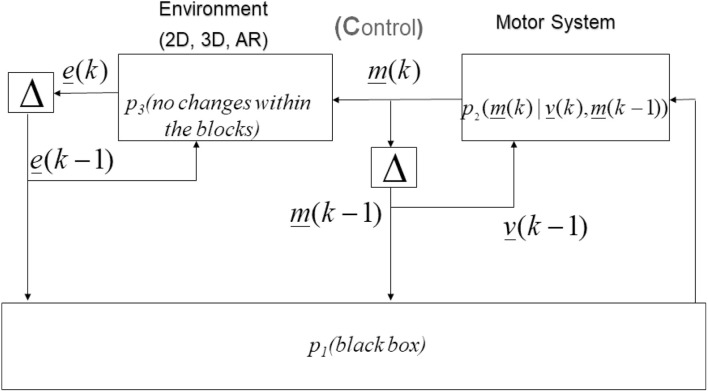
Hands motor system control driven by the environment in a Markov chain description.

Considering overall results and cognitive science application, the different 2D, 3D, AR environments (*p*_3_) have resulted in changes to motor control (*p*_2_) depending on the current motor response (*m*(*k*)) and the immediately prior motor response (*m*(*k* − *1*)), see neurocomputational model in Figure 5, extending results of Khalvati et al. (2019), Mugruza-Vassallo et al. (2021), and according to auditory results of Mugruza-Vassallo and Potter (2019). RT distribution was almost uniform across participants. Therefore those “no changes within the blocks” should be related to the environment and not necessarily to the “brain” selector inside *p*_1_ (e.g., repetitive or non-repetitive), leaving *e* (*k* − *1*) outside *p*_1_ (black box). A future experiment would be to change 2D, 3D, and AR in the same block to test how “brain” selector could change environment control.

#### Modelling limitations

The study did not use brain imaging techniques, leaving the rest of the control as a black box (*p*_1_), namely some combination of eye-tracking, EEG, and fMRI to best explain the modulation of the present findings. For example, recently it has been found that the number of prefrontal areas used during an auditory decision task was modulated by motor and inhibition response (Mugruza-Vassallo et al., 2021). Therefore, the task were mainly conducted on attention and decision-making without taking into account multitasking, leaving *p*_3_ without evidence of multimodal or multitasking distribution.

## Discussion

### Markov chains explain part of the variance in 2D, 3D, and AR

The differences observed with 2D using a Level 0 in Markov response, 3D using Level 1 and AR using Level 2 draws attention to the complexity of human reaction behaviour, i.e., RTs on accuracy classification in a visual paradigm, which is related to motor findings on auditory processing found in fMRI (Mugruza-Vassallo et al., 2021). To the knowledge of the present authors, the paradigm of Posner is the first and most common paradigm used when attempting to relate to RT in the orienting of attention paradigms. The differences observed in 2D, 3D, and AR in this study could be seen as an extension of recent neuroimaging studies, which have discussed auditory stimulus properties along with the orienting of attention, whilst considering the immediately prior stimulus (using EEG in Mugruza-Vassallo and Potter, 2019; using fMRI in Mugruza-Vassallo et al., 2021), using different regressors than visual modality (Mugruza-Vassallo, 2016b) and how visual properties can guide 2D and 3D RTs attention environments (Torres-Tejada et al., 2020). Moreover, the present authors are unaware of any articles that have sought to apply the direct choice paradigm to AR and use Markov to study cognitive human computation.

Additionally, the implications of these results may help to improve the understanding of memory in AR attentional tasks. Bearing in mind Schneider’s work (2020), it would help to develop chip devices to boost memory while working between 3D and AR. For example in Alzheimer brain processing, bearing in mind that ageing attenuates value sensitivity and integration during decision-making and requires more environmental compensation than younger adults (Chen et al., 2021) as well as extending studies of habits in executive function (Obeso et al., 2021).

### Prior state influenced RTs

The response of selective attention by means of contextual memory of a previous measure used Markov chains. The Markov chain response patterns were observed to improve when people made errors in their response to the stimuli, which resulted in RT improvement (without feedback) and dependent on the previous state. These results and the different RTs Gaussian distributions, see Table 3 for statistical means and standard deviation are consistent with different visual cognition systems as of Batallones’ findings (Batallones et al., 2015). Indeed Gaussian RTs distributions were found similar in 2D, 3D, and AR but with clearly different average RTs for AR. Batallones’ results considered the RT, however, the results of this study extend the idea to the immediately prior context of the experience affecting attentional states. Attention was studied with P300 according to the analyses in auditory cases seen with EEG (Mugruza-Vassallo and Potter, 2019) and visual in the posterior part to the pre-motor cortex seen with fMRI (Koechlin et al., 2003). Even more, the results presented here of prior state influencing RTs would be an extension to the visual environments of the anticipatory model seen in linguistics and music (Forth et al., 2016). Moreover, an extension of the analysis could be used to test for cognitive bias, for example, to avoid repetitions or random human recognition with Markov Chains (Baena-Mirabete et al., 2020). Also, our results follow and specify previous results (Soon et al., 2013), suggesting that this unconscious preparation of selective choices is not restricted to current left or right motor answer preparation. Instead, decisions in different environments (2D, 3D or AR) evolve from the dynamics of preceding cognitive activity, as shown by different Markov chains.

Alternatively, in the video game AR, the increased success when the time passes within the blocks was in line with the learning curves of the tasks (e.g., experimentally shown by Mugruza-Vassallo and Potter, 2019). This consistency is clearly related to learning the game, as the videogame is a new task the participants had to adapt to. This interpretation is may be related to a systematisation of the data, and could lead to a more precise prediction of the participants’ responses or to factors that could modulate said precision (Mugruza-Vassallo, 2016a). Improvement of prediction could allow the Go-Go attention model to be extended based on Markov decision processes that use feedback (Savalia et al., 2016). This could be achieved in three ways:

a. Adding a learning constant to the modified Bellman equation, thus adding an additional term of learning (μ),

(5) $$V^{*}\left(s_{n}\right)=\max T\left(s_{n},a,s_{n+1}\right)\left[R\left(s_{n},a,s_{n+1}\right)+\sqrt{V\left(s_{n+1}\right)+\mu V\left(s_{n+1}\right)}\right]$$

(b) Considering a reward for the previous action *R* (*n* − *1*), in which case μ = γ^2^. That is similar to the truncated n-step method proposed by Watkins, 1989, page 92). This method has recently been reconsidered in Mnih et al. (2016) and Barth-Maron et al. (2018).
(c) Reinforce Learning assumptions for the Markov decision process. Maybe the change would be the definition of the rule, which is more related to Equation 2 in a way the classic version is $\pi \left(a_{n}|s_{n}\right)$, the new version would be $\pi \left(a_{n}|s_{n},s_{n-1}\right)$. Then, re-estimate the Bellman equation.

### Markovian brain modelling on multitasking and therapy

Under the assumption of multitask reinforcement, a learning process built by two states in a Markovian tree, Tomov and colleagues provided an example of a participant going to a fast-food shop or coffee shop is a good way to gain his reward when hungry or weak or a diner shop if the participant is both hungry and weak. Moreover, the participant might find that the coffee shop has a better atmosphere or environment (Tomov et al., 2021), as here tested in a different way in 2D, 3D, and AR environments.

Figure 5 also considers some Bayesian analysis, where information on the choice of priors that best fit Markov chains, having a common path with Tomov et al. (2021). However, the present experiment was built by two states in a Markovian tree depending on the environment (i.e., 2D, 3D, and AR) with a single decision left or right. A more applied example may be when the participant has to choose the effective way to preserve his life as a reward in an earthquake, where the 2D, 3D or AR environments have equivalent but different warning signals. Therefore, warning signals on different environments might be better studied for this case to choose the route for evacuation, being the viable option about safety and the “brain” selector interaction with the environment should be further studied in the earthquake warning signals case.

An additional highlight was to corroborate how the previous response (correct or incorrect) influenced the RT to simplify the stimulus made on the experiment conducted here which: (a) complement the auditory task of Mugruza-Vassallo and Potter (2019) and 2D task of Koechlin et al. (2003); and (b) there is no bias with the type of 3D stimulus as found by Torres-Tejada et al. (2020), only for the responses presented to the prior stimulus. In this way, given that Torres-Tejada’s paradigm was simplified, it has allowed the analysis of three different scenarios, in a parallel way to auditory signals (e.g., Mugruza-Vassallo, 2016a; Mugruza-Vassallo and Potter, 2019). Moreover, when information gathered from electrodes are added to the analysis, motor hand responses produced when grasping objects and avoiding obstacles (Sun et al., 2018) would reveal different neural pathways (Mugruza-Vassallo et al., 2021). Furthermore, memory control mechanisms and their underlying brain networks could be studied together with these neuroimaging techniques to answer questions relating to resilience (such as in earthquakes) following event or object detection given by warning signals. For example, recent studies have focused on improving understanding of clinical responses and variation in response following trauma (Mary et al., 2020).

In addition, the results of the order of execution of the different environments in previous blocks (3D and AR) in the video games affected significantly for the 2D game. That is to say, the changes in stimuli were significant to the RT sequences of the 2D events which together with Bellman equation open the possibility for more experiments with other measures (eye-tracking, EEG, fMRI) and gaining a better understanding of neuronal path understanding.

### 3D and AR on gender and videogame applications

Regarding the variables of coffee, mood, and gender, gender of the participants affected the RT when the stimulus was in 3D (*p* = 0.003 @ *F* = 10.475). The foregoing is consistent with the Posner paradigm study showing how women perform endogenous visual processing tasks faster than men before turning the head 25° when the eye worked in the visual 2D environment (Cooney et al., 2017). In this way, when the stimulus presented to the right or left was evaluated and which showed significant differences between 2D, 3D, and AR, showing as a simplified response of said effects in comparison to audio-visual experiments (Córdova Berríos et al., 2018) or audio-visual including agents (Hmamouche et al., 2020).

Moreover, in AR, gender differences were not observed, meaning that exogenous AR buttons were not affected by gender. Therefore, this result is also an extension of Mitsuda et al. (2019), where exogenous processing was found different to endogenous processing using Posner’s task. Bearing in mind that during the last decade in the AR-game environment and in a therapy, the Microsoft HoloLens emulators and Unity 3D implemented a playtest environment and designed a special test environment for recent systems (Alqithami et al., 2019; Ibrahim et al., 2021). Specifically, a recent proposal for ADHD patients improved design with the measurement of the performance index (considering factors: response, impulsivity, inattention, engagement and errors throughout time slots; Alqithami, 2021). These studies would be improved with the present results bearing in mind the two previous trials on Markov chain and gender (3D vs. AR view) would improve enhancement analysis of patients’ behaviours including exogenous and endogenous interaction.

This can be interpreted as a clear difference to the bottom-up and top-down 2D mechanism interaction with the 3D and AR modalities, which would be expected in cross-modal tasks (e.g., Parmentier et al., 2010; Mayas et al., 2014; Vasilev et al., 2019; Mühlberg and Müller, 2020).

The present work should be extended, for example, to the Mario Bros video games or even comparisons between 2D and 3D (e.g., Torres-Tejada et al., 2020) extending to AR: when a third person (as stimulus) in the visual task is reached for re-identification when a participant surveys with different camera angle views (e.g., Zheng et al., 2012). An extension of the re-identification study is to understand computation of human body parts, e.g., body local representations corresponding to local similarities (Wu et al., 2018), a proposal of coattention based comparison to find best image parts correlated (Wu et al., 2021), and the difficulties in producing good feature representations (Kodirov et al., 2016) using eye-tracker could focus not only on improvements in 2D but also in 3D and AR. Therefore, interpretations could be made from cognitive human computation that other stimulus simplified was done in representation learning having the participant similar identification with all three scenarios (2D, 3D, and AR).

As an extension of videogame controlling could be explored as referred to by DeMarle (2017) and finally, the videogame reasoning here could be applied to neurogame controlling in: (a) multimodal analysis that could be extended to Cz channel laplacian (Leeb et al., 2013); or (b) machine learning processing and analysis because Petri nets use around 5 Hz steady state visual evoked potential (SSVEP) paradigm based on EEG (Sun et al., 2018).

## Conclusion

The Markov chain has confirmed the hypothesis that RTs can be predicted as a result (outputs of the states) of visual stimuli in 2D with current stimulus, 3D with current and previous stimuli, and AR with current and two previous stimuli, by means contextual memory of a previous measure for the response of selective attention in a very simplified computer game experiment designed for doing some neurophysiological recording as well.

The present study did not show significant differences in hand, mood, and coffee on a simplified videogame. On the other hand, the present study found the 3D efficacy of women was different to communicate the direction of the arrow’s attention. This extends the ongoing debate about the idea that exogenous cues affect endogenous responses in a simplified directional task. Therefore, the present results provided an insight on how exogenous AR buttons generate a different type of cueing different from 3D seen on Posner’s paradigm.

## Data Availability Statement

The datasets presented in this study can be found in online repositories. The names of the repository/repositories and accession number(s) can be found below: https://github.com/cmugruza/2D_3D_AR_rt.

## Ethics Statement

The studies involving human participants were reviewed and approved by Universidad de Lima. The patients/participants provided their written informed consent to participate in this study.

## Author Contributions

CM-V made the substantial contribution to the conception of the work and time calibration, i.e., proposed the idea of removing warning signals to seek for differences on AR, made part of data collection and children participant’s test, made the final analysis and main interpretation of data for the work, finally drafted the work and revised it critically for most of the intellectual content, made final revision, made almost all revisions from the article of the intellectual content. JG-D made a substantial contribution to the 2D and 3D design of the work, he made camera testing and part of data collection, he made the preliminary analysis, and earlier interpretation of data for the work. VF-B made time calibration of the overall experiment, made part of data collection, he helped with the preliminary analysis and he critically reviewed results and earlier discussion sections. LC-B arranged the physical initial test of the experiment, made part of data collection, she reviewed the initial draft and she critically reviewed the earlier results section. All authors contributed to the article and approved the submitted version.

## Funding

The present research experiment was in part within the project “Testeo de Electrofisiología Cognitiva de atención humana adaptado a realidad aumentada” supported by the Scientific Research Institute of the Universidad de Lima. Also, the experiment of the present research and its interpretation was part of the current analysis of the project with the support of the Research Institute of the UNTELS for financing the project “Estudio de comportamiento ocular ante sismo: escenario simulado mediante realidad aumentada”, see RCO-230-2017-UNTELS (2017–2019). Research duties provided for the first author (CM-V) under “Decreto Supremo N.° 003-2018-MINEDU,” also for some time to amend interpretation at University of Delaware under the project “EEG and fMRI studies for dyslexia” in Center for Brain and Biomedical Imaging in Delaware in 2020, and UPSJB since 2022, for overall Markov brain proposal and writing-up.

## Conflict of Interest

The authors declare that the research was conducted in the absence of any commercial or financial relationships that could be construed as a potential conflict of interest.

## Publisher’s Note

All claims expressed in this article are solely those of the authors and do not necessarily represent those of their affiliated organizations, or those of the publisher, the editors and the reviewers. Any product that may be evaluated in this article, or claim that may be made by its manufacturer, is not guaranteed or endorsed by the publisher.
