# Supplementary material for: Different Markov chains modulate visual stimuli processing in a Go-Go experiment in 2D, 3D, and augmented reality
Source: Front Hum Neurosci. 2022 Nov 21;16:955534. doi: 10.3389/fnhum.2022.955534 (PMC9769205; doi:10.3389/fnhum.2022.955534)
Supplement: Supplementary file 1 [file Data_Sheet_1.pdf]

Supplementary Material

Validation and pilot test

Before conducting the main study, a validation and a pilot test were carried out to develop and to test the videogame in all three environments (2D, 3D and AR).

Validation of experiments with RTs in UNITY

In order to validate presentation software, similar to Tejada-Torres et al. (2020), an acquisition card was used to measure the time lag that existed when recording the reaction times. To send signals to the board, a USB to TTL signal transformer was used to send as a serial port. This transformer was connected to an SCB-50 / BRD circuit board (Logicbus) and from there, an IDE cable connection to the PCI-DAS6402 receiver board (Omega Inc).
A thousand tests were made for each video game. Table S1 showed an average of 0.198 ms of lag for the 2D videogame, and a standard deviation of 1.089 ms and very similar to the 3D videogame. On the other hand, the augmented reality video game has a standard deviation of 10,483 ms.

Table S1. Times obtained by subtraction between computer processing and acquisition card in milliseconds.

	2D	3D	AR
**Average**	0.1984	0.1986	0.1706
**Standard deviation**	1.0887	1.0993	10.4382

Once the temporal resolution acquisition of the data was validated, the Markov analysis was performed. To do this, we began analysing each participant independently, then we identified the degree of influence on the reaction time of the previous states and finally the ANOVA was performed. This analysis was carried out in order to analyse whether variables such as coffee, mood and gender had affected the participant's reaction time. In the same way, its applicability was to know if the execution order of the videogames affected the reaction time of the person and finally, to analyse if previous states influenced the participant's reaction time.

Pilot Test

Pilot results of the probabilities of the 2D and 3D games of the participant were summarised in Tables S2 and S3. When the participant performed well on the first stimulus, the participant had a 96.52% chance of matching the response to the stimulus, and then came a 3.48% of probability to answer incorrectly in block 1 of the 2D game and so on with each block of each video game. Moreover, each time the participant had a 'false' answer (Table S2) then a hundred percent probability that the next stimulus will be performed correctly was found, both in 2D and 3D. On the other hand, if we take into account three orders, when having a previous FV response, in the first block of the 2D game there were no occurrences in the response of 'false'; however, in the 3D game, it was not presented in any of the three blocks.

Table S2. Probabilities to go from one state to another in order 1.

**Previous answer**	**Answer**	**2D**			**3D**		
		B1	B2	B3	B1	B2	B3
**-**	F	0,025	0,025	0,034	0,008	0,025	0,025
**-**	V	0,975	0,975	0,966	0,992	0,975	0,975
**F**	F	0	0	0	0	0	0
	V	1	1	1	1	1	1
**V**	F	0,035	0,017	0,035	0,009	0,026	0,026
	V	0,965	0,983	0,965	0,992	0,974	0,974

Table of 2D and 3D probabilities at videogame to go from one state to another in order 1.

Table S3. Probabilities to go from one state to another in order 2.

**Previous answer**	**Answe r**	**2D**			**3D**		
		B1	B2	B3	B1	B2	B3
**FF**	F	0	0	0	0	0	0
**FF**	V	0	0	0	0	0	0
**FV**	F	0	0,3333	0,25	0	0	0
**FV**	V	1	0,6667	0,75	1	1	1
**VF**	F	0	0	0	0	0	0
**VF**	V	1	1	1	1	1	1
**VV**	F	0,036	0,0088	0,0273	0,0086	0,0268	0,0268
**VV**	V	0,964	0,9912	0,9727	0,9914	0,9732	0,9732

Table of 2D and 3D probabilities at videogame to go from one state to another in order 2.

In the case of the experimental block of Augmented Reality, at the beginning the participants presented difficulties to carry out the experiment, as seen in probabilities found in each block (Table S4). For example, in block 1, there is a 6.78% chance to make a wrong answer at first stimulus; however, in blocks 2 and 3, the probability decreased to 3.3898% and indicated that the participant has improved his performance after each block. In the same way, it can be observed that, if the 'false' previous stimulus is taken, the probabilities that the current state is 'false' again, are presented only in block 1 and in the remaining blocks the probability ends up being zero percent.
Looking at Table S5, we can see that in Tables S2, S3 and S4, when you have as a previous response a 'FV' or 'VF' (first row in Table S5) the number of 'false' as current state was decreasing; this is reflected in the probabilities shown in these tables.

Table S4. Probabilities to go from one state to another in Markov chain order 1.

**Previous stimulus**		**Probability**		
		B1	B2	B3
**-**	F	0,0678	0,0339	0,0339

	V	0,9322	0,9661	0,9661
**F**	F	0,125	0	0
	V	0,875	1	1
**V**	F	0,0636	0,0351	0,0351
	V	0,9364	0,9649	0,9649

Table showing the probabilities in the Augmented Reality video game to move from a state "i" to a state "j" (for example) order 1.

Table S5. Probabilities to go from one state to another in Markov chain order 2.

**Previous answer**	**Answer**	**AR**		
		Block 1	Block 2	Block 3
**FF**	F	0	0	0
	V	1	0	0
**FV**	F	0,143	0	0
	V	0,857	1	1
**VF**	F	0,143	0	0
	V	0,857	1	1
**VV**	F	0,058	0,036	0,045

Table showing the probabilities in the 2D and 3D videogame to go from one state to the other in order 2

V 0,942 0,964 0,955

Results of the ANOVA analysis of the left/right effect in the reaction time of the laterality of the stimulus presented.

Table S7 showed the three Markov-chain models. The previous and current states have no significant effect (p>0.118 in all cases) on the reaction time on left and right responses. This means, the previous stimulus did not significantly affect a task, which suggests the effectiveness of the proposed task as a variation of the video game for selective attention without significant motor influence even when taking the previous stimuli.

Table S7.
ANOVA of the left and right stimulus effect presented and reaction times

State	Level	2D game		3D game		AR game	
		F	p	F	P	F	P
1	0	0.037	0.8491	0.000	0.9846	0.018	0.8944
2	0	0.041	0.8407	0.010	0.9192	1.409	0.2385
	1	0.004	0.9529	0.016	0.8998	0.007	0.9337
3	0	0.017	0.8966	0.018	0.8928	0.018	0.8932
	1	0.093	0.7603	0.003	0.9564	0.728	0.3947
	2	0.029	0.8644	0.045	0.8320	2.462	0.1185

In addition, in Table S8 the average reaction times between each state by presenting stimulus have shown consistency with previous works in the literature. Table S8 is showing the average reaction time between pressed stimuli. The number of occurrences (N) is also indicated

Table S8. Average reaction time between buttons pressed

**Previous state**	**State**	**2D game**		**3D game**		**AR game**	
		Average (ms)	N	Average (ms)	N	Average (ms)	N
**-**	**L**	440.38 ± 63.15	22	432.03 ± 67.64	22	867.16 ± 103.31	22
	**R**	436.76 ± 62.37	22	431.64 ± 68.01	22	863.17 ± 94.17	22
**L**	**L**	434.42 ± 62.04	22	433.75 ± 70.16	22	855.78 ± 96.64	22
	**R**	439.61 ± 61.28	22	430.33 ± 67.68	22	868.85 ± 112.17	22
**R**	**L**	437.71 ± 64.81	22	429.97 ± 70.54	22	844.67 ± 88.61	22
	**R**	437.90 ± 63.36	22	430.45 ± 64.37	22	883.61 ± 113.05	22
**LL**	**L**	437.29 ± 62.62	22	433.19 ± 75.85	22	829.77 ± 105.65	22
	**R**	453.61 ± 72.70	22	436.85 ± 71.72	22	855.98 ± 89.09	22
**LR**	**L**	422.49 ± 61.14	22	420.53 ± 65.88	22	839.52 ± 81.42	22
	**R**	434.18 ± 62.87	22	436.66 ± 73.40	22	872.42 ± 99.71	22
**RL**	**L**	439.70 ± 67.39	22	434.57 ± 66.77	22	892.19 ± 107.66	22
	**R**	426.15 ± 62.62	22	417.10 ± 58.81	22	842.41 ± 115.14	22
**RR**	**L**	453.89 ± 64.64	22	437.33 ± 60.81	22	881.63 ± 113.63	22
	**R**	434.42 ± 54.98	22	429.45 ± 75.96	22	880.81 ± 121.15	22

Our stimulus presentations has good temporal resolution in 2D and 3D presentacion, but it has temporal limitations in AR. For example, bearing in mind AR standar deviation around 10 ms, eye tracking would be find Nyquist frequency for the analysis of visual stimulus as well as to study some brain areas related to superior colliculus for a furhter use, e.g. superior colliculus brain areas that used 50 ms epoch for analysis (Nguyen et al., 2016) or their equivalent at optical tectum (e.g. at 5 ms sampling rate Fajardo, Zhu & Friedrich, 2013).

In conclusion, the present experiment and its validation has confirmed that the Markov chain hypothesis that can be predicted (RT) outputs from visual stimuli in 2D, 3D and AR States, involving contextual memory of a previous measure for the response of selective attention. Therefore, in Markov chains response patterns were observed to improve when people have errors in response to stimuli, resulting in an improvement of the response (no feedback) time dependent on the previous state. These results extend the idea of the context of the experience affects attentional (P300) State according to the analysis in cases hearing seen with EEG (Mugruza-Vassallo and Potter, 2019) and Visual on the back to the pre-motor cortex with fMRI (Koechlin et al., 2003) and even more would be an extension of the model anticipatory seen in linguistics and music (Fort et al., 2016).

Supplementary References

1. Fajardo, O., Zhu, P., & Friedrich, R. W. (2013). Control of a specific motor program by a small brain area in zebrafish. Frontiers in Neural Circuits, 7, 67.
2. Koechlin, E., & Summerfield, C. (2007). An information theoretical approach to prefrontal executive function. Trends in Cognitive Sciences, 11(6), 229-235.
3. MATLAB (1984). Obtenido de https://www.mathworks.com
4. Nguyen, M. N., Nishimaru, H., Matsumoto, J., Van Le, Q., Hori, E., Maior, R. S., ... & Nishijo, H. (2016). Population coding of facial information in the monkey superior colliculus and pulvinar. Frontiers in neuroscience, 10, 583.
5. Omega. Placa PCI en “High Speed, 16-Bit, 64 Channel Analog Input Board with Analog Outputs and Digital I/O for the PCI Bus”, Accessed on 2017, https://www.omega.com/pptst/PCI-DAS6402.html
6. Logicbus. Tabla de circuitos en “SCB-50”, Accessed on 2018, http://tienda.logicbus.com.mx/SCB-50_p_3059.html
7. Torres-Tejada S, Portilla-Fernández J.A., Mugruza-Vassallo C.A., Córdoba-Berrios L.L. (2020) Variations of reaction times explained by stimuli changes in size and perspective in 2D and 3D for selective attention. Revista mexicana de ingeniería biomédica 41(1):91–104
